# Organ donation: psychosocial factors of the decision-making process

**DOI:** 10.3389/fpsyg.2023.1111328

**Published:** 2023-05-26

**Authors:** Valeria Carola, Chiara Morale, Cristina Vincenzo, Valentina Cecchi, Livia Errico, Giampaolo Nicolais

**Affiliations:** ^1^Department of Dynamic and Clinical Psychology, and Health Studies, Sapienza University of Rome, Rome, Italy; ^2^Coordination of Organ and Tissue Donation, Anesthesia Intensive Care Unit, Sant’Andrea Hospital, Rome, Italy

**Keywords:** organ and tissue donation, psychosocial factors, clinical psychology, decision-making, communication

## Abstract

Organ donation has a crucial impact on patient care and survival, of which the worldwide gap between organ demand and supply is currently one of the most challenging issues. Brain-dead patients are the main source of organs that can be donated, but donation requires the consent of family members—a choice that is often complex and stressful and leads to refusal of consent. This mini-review aims to provide an overview of the current knowledge on the impact of certain psychosocial factors on the decision-making process with regard to organ donation by family members. In particular, the influence of several aspects is emphasized, such as sociodemographic factors, knowledge of the organ donation process, religious beliefs, concerns that are related to the choice to donate, and mode of communication. Consistent with this evidence, we emphasize the need to examine these aspects further through interventions and guidelines that improve the organ donation application process and ensure a positive experience for the family that has to make the decision.

## 1. Introduction

The intent to replace diseased or damaged body parts has been pursued for millennia throughout human history (Barker and Markmann, 2013). Egyptian, Chinese, Indian, Greek, and Roman mythology reports the practice of transferring organs or body parts from animals or a corpse to a person for healing purposes, but the first evidence of applying this method to medical practice dates to the late 19th century, when an epidermic graft by a Swiss surgeon was described (Nordham and Ninokawa, 2021).

The first successful living donor transplant of a human organ occurred only in 1954, when a boy in the US donated a kidney to his twin broche, who suffered chronic kidney failure (United Network for Organ Sharing, 2022). Subsequently, transplant procedures for other organs, such as liver, pancreas, heart, lung and intestinal organs, were performed between the 1960s and 1980s. During this time, individual hospitals handled all aspects of organ recovery and transplantation—if there was no demand in the donor’s geographic area, there was no system to locate compatible recipients, and surgeries could not be performed. Consequently, scientific organizations and a computer-based system that was dedicated to transplantation were established in the US (United Network for Organ Sharing, 2022).

According to the Italian Organ Donor Association (Associazione Italiana per la Donazione di Organi, Tessuti e Cellule, 2022), the scientific history of organ transplantation began in 1902, when the surgeon Alexis Carrel devised a technique to join 2 blood vessels. Today, the Italian National Transplant Network (Centro Nazionale Trapianti, 2022) has recorded significant increase in the rate of donations in the Italian territory, estimating 23.3 per million inhabitants in 2021. This figure is encouraging, compared with that in 2020 (20.5) and 2019 (22.8). However, the rate of opposition to postmortem organ and tissue donation is one of the main reasons why potential donors do not reach the procurement stage in Italy. The Italian system regulating organ donation is based on the “opt-out” mode, a system that moves toward the principle of “presumed consent.” All citizens over the age of 18 are automatically registered to donate their organs when they die, otherwise they must actively opt out of donation (Frati et al., 2014).

Refusal can be issued by an individual during his lifetime or later by eligible family members. In 2021, the refusal rate was 28.6%, resulting in the exclusion of 730 potential donors.

According to the European Parliament, organ donation is the act of giving organs or parts of them, without compensation, for transplantation into another person (Gruessner, 2017). With the goal of increasing the quality of life or saving the person who receives the donation, this procedure can be performed from living or deceased donors, following brain death or circulatory death for the latter (Scholz, 2020).

The European overview reveals substantial differences in the overall rates and specific types of donation between countries, although organ transplantation from deceased donors remains the most common form of transplantation throughout the EU (Vanholder et al., 2021). In this regard, the World Health Organization opines that deceased donor donation should be maximized, presenting a strategy to promote equitable access to organ, tissue, and cell transplants through voluntary donation (World Health Organization, 2019).

Despite a global decrease (−17.6%) in transplants that were performed in 2020 compared with 2019 due to the COVID-19 pandemic, a comparison of transplantation rates between the 6 WHO regions has shown that the most active region is the Americas, with 54,084 transplants (55.0 per million population), followed by Europe, with 36,181 transplants (42.7 per million population) (Global Observatory on Donation and Transplantation, 2022).

The elaborate procedure of organ donation revolves around the generosity of donors or their families, and to be successful, it needs proper interaction between the health care providers that are involved—donor hospitals constitute the springboard; thus, defining standardized evaluation criteria of their potential could promote efforts toward improving performance (Castillo-Angeles et al., 2021).

Considering the gap between the demand and supply of donor organs as a major global concern (Birtan et al., 2017), the characteristics of the organ donation process must be examined, and the psychosocial factors the influence decision-making should be defined. Understanding that brain-dead people are the main source of organs for patients who are in need of transplants, the decision to donate their organs is difficult and can be traumatic for family members (Ahmadian et al., 2020). In the complex process of organ donation, underestimating the emotional responses of donor family members and failing to provide clear information on brain death and donation procedures can completely inhibit conscious choices (Bocci et al., 2016).

Building on available evidence (Birtan et al., 2017; Ahmadian et al., 2020; Castillo-Angeles et al., 2021), several factors intervene in family decision-making: religious, cultural, and social beliefs; concerns over the patient’s death and the integrity of the body after death; and certain sociodemographic variables of family members. Our aim was thus to examine the psychosocial factors that are involved in organ donation.

## 2. Methods

We performed a literature search of studies on organ donation and transplantation, focusing on the psychological factors that are involved in the decision-making process. The studies were selected, based on the following topics: psychological impact of the request to donate organs, development of organ donation process over the years, mode of communication in the hospital setting, and the experiences of family members who consented to or refused organ and tissue donation.

Global databases, such as PsychInfo, PubMed, and Google Scholar, were queried, using the following keywords: organ donation, communication, brain death, loss experience, guidelines, organ and tissue donation request, and family. Through a manual search, more relevant articles were selected, including systematic reviews, research reports, and case reports. Most of the studies were published between 2012 and 2022 (Table 1).

**Table 1:** Selected studies on organ donation and transplantation, focusing on the psychological factors that are involved in the decision-making process.

Study, year	Country	Study design	Population	Methods	Outcomes
Zhang et al. (2017)	China	Cross-sectional study	Transplant patients and caregivers (*N* = 426)	Survey	**More knowledge about OD leads to greater propensity to donate.** Transplantation patients are more willing to donate organs after death than their caregivers. There is a significant relationship between participants’ willingness and knowledge of organ donation; patients with more understanding of the transplantation and donation procedure were more willing to donate organs after death.**Religious beliefs affect willingness to donate.** Moral conflict with some Confucian values represents an obstacle to consent to OD.
Ahmadian et al. (2020)	Iran	Inductive qualitative design study	Family members of brain-dead patients (*N* = 17 family members of *N* = 11 patients), medical staff (*N* = 5)	Semi-structured interviews	**Exploration of stressors**. Six themes explain the complexity of decision making: perceived threat of loss, decision making under conflict, painful corrosive farewell, a feeling of insecurity, complexity of grief, and seeking relief.
Bocci et al., 2016	Italy	Single-center study	Family members of brain-dead patients (*N* = 291)	New communication protocol	**Better medical staff-family communication protocols increase consent to donations.** Highlight on empathy and systematic introduction of the protocol was associated with a nearly 2-fold decrease in the rate of donation refusals.
Ruta et al. (2021)	Italy	Interventional quantitative study	Adult citizens (*N* = 281)	Surveys; education programmes	**More knowledge about OD leads to greater propensity to donate**. Participants appreciated the increase in their knowledge, and many filled out donor cards.
Lim et al. (2020)	Malaysia	Cross-sectional analytical study	Outpatient clinic patients (*N* = 383)	Survey	**More knowledge about OD leads to greater propensity to donate. Sociodemographic factors are associated with propensity to OD.** 5 main factors influencing the attitude toward organ donation among patients were identified: education level, occupation, monthly income, ethnicity, and knowledge regarding organ donation and brain death.
Caballero et al. (2012)	Spain	Single-center study	Family members of brain-dead patients (*N* = 52)	New communication protocol	**Better medical staff-family communication protocols increase consent to donations**. **New clinical guidelines for medical staff-family interviews**. Clinical guidelines were implemented and decreased organ donation consent refusals.
Dos Santos et al. (2014)	Spain	Qualitative study	Family members of brain-dead patients (*N* =10)	Semi-structured interviews	**Better medical staff-family communication protocols increase consent to donations. More knowledge about OD leads to greater propensity to donate.** Factors that can hinder consent: experiences related to the loss and the grieving process, lack of knowledge of the aspects relating to OD, the perception of the care provided to the patients.
Ríos et al. (2020)	Spain	Cross-sectional study	Adult citizens (*N* =3371)	Questionnaires	**Religious beliefs affect willingness to donate.** 53.7% of Catholics were in favor of OD compared with 25.4% of Muslims; the attitude toward OD is more positive in those surveyed who believe that their religion is in favor compared with those who think that their religion is not in favor of it (48% vs 5%).
González et al. (2021)	Spain	Qualitative and quantitative study	Adult citizens (*N* = 1065) for surveys, interviews of families of potential donors (*N* = 1158) for qualitative analysis	Survey and interviews	**Exploration of stressors.** The factors related positively to be a donor were being younger, completing higher studies, having talked to family about their wish, accepting a relative’s organ donation, and knowing the “opt-out” model of the Spanish donation system. Reasons to decline OD: suspicion of refusal in life of the possible donor (34.7%), the desire to maintain the integrity of the body (32.6%), whereas disinformation about donation process and the consideration that brain death does not mean the death of the individual were the less chosen options (0.8% and 1.7%, respectively). Solidarity was the main reason to accept OD (84.4%).
Emiral et al. (2017)	Turkey	Questionnaire validation study	Nonmedical staff members (*N* = 540)	Survey	Organ-Tissue Donation and Transplantation Knowledge Scale (ODTKS) validated
Birtan et al. (2017)	Turkey	Descriptive study	Family members of brain-dead patients (*N* = 12)	Surveys and interviews	**Exploration of stressors. Religious beliefs affect willingness to donate**. Breaching the body integrity, not knowing the wish of the deceased, little knowledge of brain death are factors that hinder consent.
Can and Hovardaoglu (2017)	Turkey	Retrospective study	Family members of brain-dead patients (*N* = 101)	Survey	**Exploration of stressors. Better medical staff-family communication protocols might increase consent to donations.** Strongly related with the family decision, are the wishes of the deceased persons about donation, suspicions regarding brain death, the desire to protect body integrity, and the satisfaction levels of the families with the approaches of medical personnel.
Leblebici (2021)	Turkey	Single-center retrospective study	Family members of brain-dead patients (*N* = 82)	Surveys and medical records	**Religious beliefs affect willingness to donate**. Distrust in the health care system had a significant impact on refusals to consent as well as the age of the deceased (more likely if a child rather than an adult) and causes of death (more likely if encephalitis rather than nontraumatic intracranial hemorrhage).
Sque et al. (2018)	United Kingdom	Explorative qualitative study	Family members of brain-dead patients (*N* = 43 relatives of *N* = 31 patients)	Semi-structured interviews	**Better medical staff-family communication protocols increase consent to donations.** Positive family care experience is associated with increased donation approval. The aspects connected to the temporality of the interventions should be further investigated.
Ma et al. (2021)	United Kingdom, Australia; Brasil; France, Iran; Norway	Qualitative systematic review	Scientific publications (*N* = 6)	Semi-structured interviews	**Exploration of stressors.** Three themes identified: Ambivalence due to the Ambiguity of Brain Death, Uncomfortable donation requirement conversation, Support needed after donation
Castillo-Angeles et al. (2021)	/	Systematic review	Scientific publications (*N* = 72)		**Better medical staff-family communication protocols increase consent to donations.**
de Moraes et al. (2018)	/	Reflective trial	Scientific publications (*N* = 36)		New clinical guidelines for medical staff-family interviews.

## 3. Results

### 3.1. Sociodemographic factors

Sociodemographic factors, such as age, gender, ethnicity, education level, monthly income, and cause of death, influence the propensity of a potential donor/family member to consent to organ donation (Emiral et al., 2017; Zhang et al., 2017). In particular, age is discussed extensively, because several studies (Emiral et al., 2017; Zhang et al., 2017; Lim et al., 2020; Leblebici, 2021) have reported conflicting results on its impact shows a higher likelihood of family consent to organ donation in adults than in deceased children and in older children than in younger children. Lim et al. (2020), on the other hand, showed that there were no significant associations between age and respondents’ attitudes toward organ donation. In contrast, marital status (Lim et al., 2020), length of stay in the Intensive Care Unit (ICU), and other aspects, including alcohol and substance use, do not appear to have a significant impact (Lim et al., 2020; Leblebici, 2021).

### 3.2. Knowledge of the organ and tissue donation process

Several studies have focused on family members’ understanding of the meaning of brain death (Lim et al., 2020), their knowledge of the organ donation process (Lim et al., 2020), and their perceived confidence in the medical care that is provided to their family member (Bocci et al., 2016; Birtan et al., 2017; Ahmadian et al., 2020; Leblebici, 2021; Ruta et al., 2021). These factors are central in the decision-making process that the potential donor’s family member must contemplate.

Brain-dead people are often the main source of organs (Ahmadian et al., 2020); thus, family members often receive a request to donate organs when they are informed of brain death and, consequently, beginning the grieving process (Ahmadian et al., 2020). Separating the 2 conversations and providing accurate information appears to be appropriate in facilitating the decision-making process (Bocci et al., 2016). Several studies indicate that understanding brain death increases the capacity of a family member to accept such a loss (Birtan et al., 2017; de Moraes et al., 2018).

Conversely, the lack of deep knowledge of brain death, coupled with the emotionally stressful impact of such news, can foster illusory hopes in a family member (Ahmadian et al., 2020). Accepting brain death as certain death (Birtan et al., 2017; Ahmadian et al., 2020) is complex, because somatic death does not occur. The thoughts, beliefs, and images that are usually associated with the concept of death differ (Leblebici, 2021). In this regard, the concept of death in the minds of family members does not coincide with medical criteria, complicating the already extremely difficult decision-making process (Ma et al., 2021). The most immediate image of death is evoked by cardiac and respiratory arrest and, most importantly, a cold body (Can and Hovardaoglu, 2017). Brain death differs and is often confused with a vegetative state and coma (Can and Hovardaoglu, 2017).

Concerns over respecting and maintaining the dignity of the body of a brain-dead loved one (Sque et al., 2018; Ahmadian et al., 2020) and over the perception of the medical care that is received by one’s family member (Can and Hovardaoglu, 2017) also shape organ and tissue donation. Deficiencies in medical care or perceived errors by family members create feelings of insecurity and hinder decision-making (Ahmadian et al., 2020). Thus, providing information on medical history and the process of organ and tissue donation is necessary (Dos Santos et al., 2014). In general, knowledge of organ and tissue donation is associated with a greater propensity to donate (Zhang et al., 2017; Ruta et al., 2021).

### 3.3. Religious beliefs

Religious, cultural, and social beliefs are important factors in the decision-making process for family members who are asked to donate their loved ones’ organs (Bocci et al., 2016; Birtan et al., 2017; Ahmadian et al., 2020). Religious beliefs over organ donation vary between religions (Leblebici, 2021), but in general, their influence is significant (Can and Hovardaoglu, 2017; Leal de Moraes et al., 2019). For example, religious beliefs often contradict the concept of brain death. It is common for this aspect to be associated with a belief in maintaining the integrity of the body after death (Birtan et al., 2017; Can and Hovardaoglu, 2017). In fact, the interpretation of religious principles can lead people to believe that the body should not be manipulated. The inviolability of the body may be a condition for resurrection, which is often why people refuse organ and tissue donation (Leal de Moraes et al., 2019). Fatalism is another central factor and is linked to the belief that all events have been predetermined. In this sense, organ donation opposes the will of the divine creator (Can and Hovardaoglu, 2017).

### 3.4. Concerns related to choice

The decision-making process activates internal conflict that assumes the form of an ethical dilemma. Through donation, family members may feel that the death of their loved one was not in vain (Sque et al., 2018). Yet, family members might feel that they are taking on tremendous responsibility at an already emotionally stressful time (Ruta et al., 2021) and are called on to do so in a significantly shorter period than the time it takes to grieve (Can and Hovardaoglu, 2017). Among several variables, time is important (Dos Santos et al., 2014; Can and Hovardaoglu, 2017) in influencing a family’s attitude toward this decision.

Solidarity (González et al., 2021) and the belief that they are acting in accordance with the personality of a loved one who was often attributed such character traits as “generous” and “kind” are variables that influence the choice to donate organs (Sque et al., 2018). The state of shock, time pressure, and the request for organ donation create a complex emotional experience for the family who experiences a loss. In this sense, concern over judgment by others is another variable that affects decision-making (Ahmadian et al., 2020). In fact, the complex family dynamics that arise could hinder decision-making.

Knowing the deceased person’s wishes helps a family in the decision to donate organs (Sque et al., 2018). A family that is aware of a deceased person’s wishes respects them. In contrast, not knowing such intentions more often orients the family toward refusal (Can and Hovardaoglu, 2017).

Thoughts regarding the burial of a loved one are another concern in the choice to donate organs (Ahmadian et al., 2020). Family members may feel confused, given the uncertainty of the timing and possible changes to the family member’s body (Birtan et al., 2017; Ahmadian et al., 2020).

### 3.5. Mode of communication

Brain death can occur suddenly and unexpectedly. Family members are often asked to make the decision to donate organs quickly, at an already complicated time (Ahmadian et al., 2020; Ma et al., 2021). Yet, communication is often perceived by family members as inadequate, unclear, and inappropriate—i.e., when they are not ready to discuss it (Bocci et al., 2016; Sque et al., 2018; Ma et al., 2021). Thus, the mode of communication that a professional chooses with family members assumes a central and significant role in the decision-making process. Families who are able to discuss organ donation topics qualitatively more with health professionals are more likely to consent to donation (Bocci et al., 2016; Castillo-Angeles et al., 2021). Similarly, there is a correlation between satisfaction and positive experiences with family care and subsequent consent to donate (Sque et al., 2018).

The location where the communication takes place, the participants, and verbal and nonverbal expressions are important communication-related factors (Bocci et al., 2016; de Moraes et al., 2018).

Family members need clear and comprehensive information (Bocci et al., 2016; Birtan et al., 2017) and empathic and sensitive communication (Dos Santos et al., 2014; Can and Hovardaoglu, 2017), and when they fail to be conveyed, a lack of trust in the health care team can lead to uncertainty over the quality of care that is provided to their relatives, especially in the case of organ donation refusal (Ahmadian et al., 2020). Professionals who inform relatives of a death, for example, should use such terms as “deceased” instead of “patient” (Caballero et al., 2012; Ahmadian et al., 2020) and “death” instead of “brain death”, to facilitate the process of accepting death.

Several studies (Ma et al., 2021) emphasize the desire of family members to have access to psychological support following organ and tissue donation of their loved ones. Psychological support is needed not only during this difficult decision but also, and especially, postdonation, both short term and long term.

A follow-up meeting could provide clarification to families and give updates on the health of the recipients of their family members’ organs (Ahmadian et al., 2020; Ma et al., 2021).

## 4. Discussion

Several groups (Emiral et al., 2017; Zhang et al., 2017; Lim et al., 2020; Leblebici, 2021) have examined the function of sociodemographic factors in the process of organ donation. Gender and age do not impact the level of knowledge of this process (Emiral et al., 2017), but such data appear to contribute to the likelihood of consenting to donation. Compared with men, women are more likely to donate their organs after death (Zhang et al., 2017), but some studies (Lim et al., 2020; Leblebici, 2021) have reported that sex is not a relevant factor in decision-making. In contrast, age is a controversial factor, some studies (Lim et al., 2020) claiming that it is not associated with consent to donate, whereas others have highlighted its importance. Certain groups (Zhang et al., 2017) suggest that individuals with a higher age are less willing to donate organs, and others (Leblebici, 2021) have focused on the characteristics of the potential donor, implicating the patient’s age as a determinant of the choice of the family, wherein consent is more frequent for adult decedents than for children and for older versus younger children.

With regard to other sociodemographic factors, a higher level of education and higher income are significantly associated with a positive attitude toward organ donation (Lim et al., 2020), whereas ethnicity is an underrepresented factor.

Another psychosocial factor that influences decision-making in organ donation is knowledge of the donation process (Bocci et al., 2016; Ahmadian et al., 2020; Lim et al., 2020; Leblebici, 2021; Ma et al., 2021). Such knowledge eliminates erroneous assumptions and improves consent for organ donation by patients and family members (Emiral et al., 2017; Zhang et al., 2017; Ruta et al., 2021).

Given that brain-dead people are often the main supply of organs (Ahmadian et al., 2020), it would be advantageous to promote knowledge on brain death of the potential donor (Bocci et al., 2016; Birtan et al., 2017; de Moraes et al., 2018). The families of these patients might fail to differentiate brain death and the functioning of the rest of the body, necessitating information to understand the relative’s condition and begin the grieving process (Can and Hovardaoglu, 2017; Sque et al., 2018).

Religious beliefs, as a system of values, have a significant impact on the choice of organ donation (Birtan et al., 2017). According to Leblebici (2021), religious concerns are one of the most influential factors in refusing to donate organs (Ríos et al., 2020) compared the attitudes of Catholic and Islamic populations toward organ donation, finding that the Catholic religion was a predisposing factor for organ donation. In general, if subjects believe that donation is congruent with the values of their religion, they are much more likely to have a positive opinion of donation. This result was replicated by Can and Hovardaoglu (2017), who highlighted how brain death and the integrity of the body are elements that contradict the values of religions, generating a moral conflict, as seen in other studies (Birtan et al., 2017; Leblebici, 2021). These studies thus suggested that promoting organ donation among religious leaders could be an important tool.

The world’s greatest religions (Christianity, Judaism, Hinduism, Buddhism, Confucianism) highlight different understandings of the body, death, and organ transplantation.

The Christian faith seems to approve of organ transplantation, which is seen as a personal choice. Jehovah’s Witnesses, distinct from mainstream Christianity, show a different and more complex conception, compounded by the rejection of blood transfusion. The possibility of free choice on the issue of organ transplantation for Jehovah’s Witnesses came in the 1980s, while retaining the blood bond (Oliver et al., 2011).

The Jewish faith embraces a complex debate: avoiding any unnecessary interference with the copre after death is one of the most significant principles because it allows for proper burial. At the same time saving lives is one of the major commandments. “Goses” is an Alachic term describing a sick person at risk of death within 3 days. In this case, the Jewish faith seems to reject interference-for example, medically intervening to prepare him or her for organ donation-so as not to hasten death (Oliver et al., 2011).

Similarly complex appears the Buddhist conception in which selfless giving corresponds to a central principle. However, the concept of brain death is controversial because in some Buddhist traditions spiritual “consciousness” can remain in the body for days after the exhalation of the last breath. Interfering with the dying process could therefore negatively interfere with the person’s rebirth.

Confucianism requires respecting the body (hair and skin) from birth to death because they are considered gifts from the parents. Donating organs would therefore not be respectful to the parents (Oliver et al., 2011).

The conception of the body and organ donation in Hinduism appears to be different. Physical integrity is not necessary for the reincarnation of the soul, a founding belief for Hindus. Helping those who suffer and donating (Daan) is part of virtuous acts (Niyama) (Oliver et al., 2011).

As discussed, one of the most intricate aspects is the complex emotional management of grief and the urgency of making a decision regarding organ donation, which is governed by many intervening factors. Two of the most frequent concerns are the time that given to the family for making a decision and the appropriate timing for interviewing families about organ donation by a deceased relative (dos Santos et al., 2014). A short period for responding to a request for organ donation steers families toward a negative decision, whereas a longer time is more conducive for a positive response (Can and Hovardaoglu, 2017; Ma et al., 2021). The meaning that is attributed to the gesture of organ donation by family members has emerged as an important element, which is contested by predisposing and deterring factors. The literature highlights situations in which organ donation is a prosocial behavior, for which the possibility exists of “bringing out something positive from a very negative situation” (Sque et al., 2018), “making people happy who they were still hopeful” (Can and Hovardaoglu, 2017), and being in solidarity (González et al., 2021)—thus imagining organ donation as an event that triggers positive events for themselves and the community of reference and seeking recognition from the community (Sque et al., 2018). Conversely, there are concerns over judgments that other people might have on giving consent (Ahmadian et al., 2020). However, the literature is consistent, in that knowledge of the deceased person’s desire is a facilitating factor in making a decision, especially if the deceased expressed himself positively (Birtan et al., 2017; Can and Hovardaoglu, 2017; de Moraes et al., 2018; Sque et al., 2018; Leblebici, 2021; Ruta et al., 2021).

Ultimately, the literature suggests that a first prerequisite for donation is to first inform the population about the possibility of donation in general. Organ donation protocols should aim at organizing times, spaces, and methods of communication to ease the stress, pain, and pressure that family members experienced at the time of communication.

Several studies (Can and Hovardaoglu, 2017; Ahmadian et al., 2020) show that a negative decision by the family is traced to the inability of health care professionals to communicate empathetically and sensitively and to a lack of trust in the care that is delivered to family members.

Some authors (Kesselring et al., 2007) divide the behavior of health care professionals who are involved in organ donation into 2 categories: organ-centered and individual-centered. In the former, behavior is limited to applying medical procedures to request and protect organs, whereas in the latter, professionals accommodate the family’s needs for care, time, silence, and clarification with a clear and empathic mode of communication. The authors (Kesselring et al., 2007) point out that families reported having more positive experiences when they felt that they received care and attention from health professionals. In the organ-centered approach, however, families may report traumatic experiences and negative outcomes.

These data are consistent with a review (Castillo-Angeles et al., 2021) that highlighted the need to use patient-centered and family-centered interventions. Staff training, donation requests from a trained professional, and family support in the ICU can positively influence family members’ decisions (Bocci et al., 2016; de Moraes et al., 2018; Castillo-Angeles et al., 2021).

Most of the considered literature converges on the retrospective (Can and Hovardaoglu, 2017; Sque et al., 2018) and review survey (Castillo-Angeles et al., 2021; Ma et al., 2021). Several studies have progressed toward establishing a protocol (Caballero et al., 2012; Bocci et al., 2016; de Moraes et al., 2018).

## 5. Future perspectives

This review has identified psychosocial factors that are significantly involved in the organ and tissue donation decision-making. Knowing the wishes and beliefs of loved ones is a central factor that facilitates the decision-making process and relieves family members of an emotionally and ethically onerous choice. Therefore, though research on this issue is virtually non-existent, studies on the emotional reactions and adjustment of family members involved in the donation process are highly recommended and needed.

It would also be desirable to raise awareness and promote public education on organ donation to overcome false myths and misconceptions, given that more knowledge on this topic is associated with a greater propensity to donate. In particular, future studies should address new ways of communicating with the patient’s family, an aspect that has been poorly examined in Italy. Further research should determine the impact of various educational and communication strategies with patients’ families. These findings support the need to develop an efficient protocol for organ and tissue donation, providing specific training for health care workers who are involved in the donation process and psychological support for donor families.

## Author contributions

CM, CV, and VnC performed the bibliographic search. All authors contributed to the composition of the manuscript.

## Conflict of interest

The authors declare that the research was conducted in the absence of any commercial or financial relationships that could be construed as a potential conflict of interest.

## Publisher’s note

All claims expressed in this article are solely those of the authors and do not necessarily represent those of their affiliated organizations, or those of the publisher, the editors and the reviewers. Any product that may be evaluated in this article, or claim that may be made by its manufacturer, is not guaranteed or endorsed by the publisher.
